# Study protocol for the TROG 21.08 VITaL trial: a randomized controlled trial investigating ventilation imaging to improve the quality of life for patients with lung cancer treated with radiation therapy

**DOI:** 10.1016/j.ctro.2026.101312

**Published:** 2026-10-08

**Authors:** Shalini K. Vino d, Val Gebski, Sashendra Senthi, Cynleen Kai, Dasantha Jayamanne, David Palma, Nicholas Hardcastle, Hilary L. Byrne, Douglas A. Hoover, John Kipritidis, Anthony Espinoza, Benjamin Zwan, Guneet Kaur, Amit Sawant, Bilal A. Tahir, Rebecca Mercieca-Bebber, Karan K. Shah, Andrew Bowen, Miriam Cavanagh, Lee Hunt, Surein Arulananda, Helen Ball, Nina Eikelis, Alisha Moore, Shona Silvester, Paul Keall

**Affiliations:** a Cancer Therapy Centre, Liverpool Hospital, South Western Sydney Local Health District, NSW, Australia; b South West Sydney Clinical Campuses, School of Clinical Medicine, Faculty of Medicine and Health, University of New South Wales, Sydney, NSW, Australia; c Sydney School of Public Medicine & Health, University of Sydney, Sydney, NSW, Australia; d Collaboration for Cancer Outcomes Research and Evaluation (CCORE), Ingham Institute for Applied Medical Research, Liverpool, NSW, Australia; e Sydney Clinical Trials Centre, Faculty of Medicine and Health, University of Sydney, Sydney, NSW, Australia; f Alfred Health Radiation Oncology, Melbourne, VIC, Australia; g Central Clinical School, Monash University, Melbourne, VIC, Australia; h Austin Health, Melbourne, VIC, Australia; i Northern Sydney Cancer Centre, Royal North Shore Hospital, Sydney, NSW, Australia; j Genesis Care, Sydney, NSW, Australia; k Sydney Medical School, University of Sydney, Sydney, NSW, Australia; l Verspeeten Family Cancer Centre, London Health Sciences Centre, London, Ontario, Canada; m Division of Radiation Oncology, Western University, London, Canada; n Department of Radiation Oncology, Peter MacCallum Cancer Centre, Melbourne, VIC, Australia; o Sir Peter MacCallum Department of Oncology, University of Melbourne, Melbourne, VIC, Australia; p Image X Institute, Sydney School of Health Sciences, Faculty of Medicine and Health, University of Sydney, Sydney, NSW, Australia; q Central Coast Cancer Centre, Gosford Hospital, Gosford, NSW, Australia; r School of Information and Physical Sciences, The University of Newcastle, Newcastle, NSW, Australia; s Department of Radiation Oncology, University of Maryland School of Medicine, Baltimore, MD, USA; t POLARIS, Section of Medical Imaging and Technologies, Division of Clinical Medicine, School of Medicine and Population Health, University of Sheffield, Sheffield, United Kingdom; u Insigneo Institute for in silico Medicine, University of Sheffield, Sheffield, United Kingdom; v Thoracic Oncology Group of Australasia, Thornbury VIC, Australia; w Faculty of Medicine and Health, University of New South Wales, Sydney, NSW, Australia; x Cancer Voices NSW, Milsons Point, NSW, Australia; y Department of Medical Oncology, Monash Health, Melbourne, VIC, Australia; z School of Clinical Sciences, Monash University, Melbourne, VIC, Australia; aa 4DMedical, Melbourne, VIC, Australia; ab Trans-Tasman Radiation Oncology Group, Waratah, NSW, Australia

**Keywords:** Quality of life, Lung cancer, Healthy lung-sparing, Functional imaging, CT ventilation imaging, Pneumonitis

## Abstract

**Background:**

Radiation therapy (RT) is indicated for 77% of patients with lung cancer. Over 20% of patients are affected by radiation-induced lung injury. Computed tomography ventilation imaging (CTVI) estimates high and low functioning lung regions. During RT planning, CTVI can guide preferential avoidance of higher ventilated regions by redirecting dose to less well ventilated regions, sparing healthy lung tissue. The double-masked randomized Trans-Tasman Radiation Oncology Group (TROG) 21.08 VITaL trial will investigate if sparing healthy lung can improve the quality of life (QoL) for patients with stage 3 non-small cell lung cancer (NSCLC) treated with RT.

**Methods:**

165 patients will be recruited to test the hypothesis that patients receiving healthy lung-sparing RT have better QoL than patients receiving standard RT. CTVI will be generated from exhale and inhale breath-hold CT scan pairs. A healthy lung-sparing plan and a standard treatment plan will be created for each patient and randomly assigned in a 1:1 ratio for the patient's treatment. QoL will be assessed with the Functional Assessment of Cancer Therapy-Lung questionnaire before and after treatment. Secondary hypotheses include that patients in the healthy lung-sparing arm will have fewer treatment side effects, better lung function, be more likely to receive and complete adjuvant immunotherapy, and healthy lung-sparing treatment will be cost-effective. Patient follow-up will continue for three years.

**Discussion:**

This prospective randomized trial will determine whether the incorporation of healthy lung-sparing RT based on CTVI improves QoL, reduces pulmonary toxicity, and increases eligibility to receive curative immunotherapy for patients with stage 3 NSCLC.

**Trial Registration:**

ClinicalTrials.gov (NCT06127654).

## Highlights

• Phase III randomized trial evaluating healthy lung-sparing radiotherapy.
• CT ventilation imaging enables preferential avoidance of highly functional lung regions.
• VITaL tests whether lung-sparing radiotherapy improves patient-reported quality of life.
• Secondary endpoints include toxicity, lung function, immunotherapy completion, and cost-effectiveness.
• Results will provide high-level evidence for functional imaging-guided radiotherapy in stage III NSCLC.

## 1 Introduction

Lung cancer, the leading cause of cancer mortality and disability-adjusted life years in the world [1] has a 5-year survival rate of only 28% [2]. Radiation therapy (RT) will benefit 77% of lung cancer patients and plays a central role as curative treatment in unresectable stage 3 NSCLC [3]. RT carries risks, including radiation-induced lung injury, deteriorating QoL [4] and survival [5]. Pneumonitis is the most common radiation therapy side effect, occurring more frequently in patients with stage 3 NSCLC receiving adjuvant immunotherapy where grade 2+ pneumonitis rates range from 23%–44% [6], [7], [8], [9]. A multicenter study found 86% of pneumonitis was attributable to RT, with 11% related to both RT and immunotherapy [8].

Pneumonitis impacts the ability to receive adjuvant immunotherapy in locally advanced NSCLC, a treatment with significant survival benefit [10]. Pneumonitis is the most common reason for not commencing, interrupting or ceasing immunotherapy early with 10–15% patients discontinuing adjuvant immunotherapy due to pneumonitis [8], [9], [11] resulting in poorer survival [12], [13]. Xu et al. reported a reduction in the four-year overall survival from 60.2% for patients completing immunotherapy to 45.4% if immunotherapy was ceased due to pulmonary toxicities [13].

The planning and delivery of RT is a balance between delivering a curative dose to the tumor while sparing healthy organs, such as the lungs, from side effects including pneumonitis. To minimize radiation-induced lung injury, spatial mapping of lung function using functional imaging modalities has been used as the basis for directing radiation away from the healthy, high-functioning regions towards the low functioning regions with the intent of minimizing post-RT loss of respiratory function, and thereby improving the patient's QoL [14].

CTVI shows high and low functioning lung regions overlaid on an anatomical CT image and can generate the ventilation images from CT scans routinely acquired for lung cancer RT planning [15]. Other modalities for lung ventilation imaging include xenon CT, single photon emission CT, positron emission tomography (PET), and hyperpolarized gas magnetic resonance imaging [14], [16], [17]. One systematic review [18] (69 included studies) found moderate to strong evidence that CTVI provides the same regional ventilation information as these other modalities. A second systematic review [16] (114 included studies) proposed that further clinical studies were needed to detect differences between the anatomic and functional dose metrics. A recent systematic review and meta-analysis on the incorporation of functional lung imaging into RT planning found a 13% (95th percentiles 8%–20%) rate of grade 2+ radiation pneumonitis, with functional avoidance planning [14]. The review concluded that interventional trials related to functional lung avoidance planning are sparse, but are critical to understanding the effects on reducing toxicity and improving patient outcomes.

Several prospective and retrospective CTVI studies have shown that sparing highly ventilated regions of the lung may reduce treatment-related toxicity [19], [20], [21], [22], [23], [24], [25], [26], [27], [28], [29]. A summary of three prospective CTVI trials is given in Table 1. CTVI is low cost, easily implemented into the treatment workflow, and could therefore be made widely available. The phase 3 double-masked randomized controlled VITaL trial will provide level 2 evidence to inform clinical practice.

**Table 1:** Prospective CTVI trials that informed the VITaL phase 3 trial. PET = positron emission tomography. RT = radiation therapy.

Trial ID	Sponsor	Description/Primary endpoint	Relevant finding
NCT02528942	University of Colorado	67-patient trial assessing safety and feasibility of CTVI RT (grade 2+ radiation pneumonitis)	CTVI is convenient. A phase 3 trial is warranted [25]
NCT02308709	University of California-Davis	34-patient trial assessing safety and feasibility of CTVI RT (grade 3+ adverse events)	CTVI functional avoidance RT with significantly reduced dose to ventilated lung regions could be safely administered [28]
NCT02843568	University of Wisconsin	122-patient trial assessing CTVI and lung tissue changes pre/post RT	Dose to healthy lung is associated with pneumonitis, RT should limit dose to functional regions [26], [29]

## 2 Methods/design

### 2.1 Study design

The study is a multicenter, prospective, double-masked, randomized controlled, two-arm, phase 3 trial, that will be conducted initially in Australia and then extended internationally. The sponsor is the University of Sydney. TROG is providing trial credentialling and quality assurance services.

### 2.2 Objectives

The trial will test whether patients receiving healthy lung-sparing RT will have better QoL at 3 months post-treatment than patients receiving standard RT. Secondary hypotheses include that patients in the healthy lung-sparing arm will have fewer treatment-related side effects, better lung function, and be more likely to receive and complete post-RT immunotherapy. A health economics assessment will provide critical context to the clinical findings.

### 2.3 Eligibility criteria

Eligible participants will be adults over 18 years, have Eastern Cooperative Oncology Group (ECOG) performance status ≤2, have histologically proven stage 3 NSCLC (following the International Association for the Study of Lung Cancer (IASLC) 8th edition lung cancer staging guidelines), and will be treated with curative intent with external beam RT (60 Gy in 30 treatment fractions or 55 Gy in 20 treatment fractions) with or without concurrent chemotherapy and adjuvant PD-1/PD-L1 inhibitors. Participants will be willing and available for informed consent, visit requirements, and follow-up. Subject to availability, translators will be used to assist patients. Participants will have a PET scan within eight to twelve weeks of registration, and pulmonary function tests within twelve weeks of registration. Exclusion criteria include a diagnosis of interstitial lung disease, a prior history of lung RT, a prior treatment for this cancer with surgery within a year or systemic chemotherapy for this cancer.

Post-inclusion criteria for the VITaL trial, which are not known prior to consent, are:

1. FACT-L questionnaire Trial Outcome Index (FACT-L TOI) score of ≥5, the clinically meaningful difference [30].
2. The standard treatment plan shows that at least 16% of the volume of the lungs (minus the Gross Tumor Volume) will receive more than 20 Gy. This criterion is based on the difference in pneumonitis risk between patients receiving below (∼8%) and above (∼34%) the 20 Gy threshold [31].
3. The total lung volume difference between the inspiratory and expiratory CT images must be more than 500 cc, or more than 10% of the expiration CT volume, a requirement of the CT:VQ product (4D Medical, Melbourne, Australia) that is used to create the ventilation images.

Ineligible patients will be informed and removed from the trial. Their treatment will follow the treating center's standard of care.

### 2.4 Interventions

The VITaL trial schema is shown in Fig. 1. For all patients, after consent and during the normal pre-treatment CT imaging session, additional breath*-*hold inhale and exhale CT scans will be acquired. From the breath*-*hold CT images, a CT ventilation image will be created using the CT:VQ product and imported into the treatment planning system. Two treatment plans will be created for each patient: a healthy lung-sparing plan and a standard treatment plan. The healthy lung sparing plan will use the ventilation image to create an RT plan with lower dose to the high functioning lung compared to the standard RT plan. A quality assurance process has been developed to balance the mean lung dose and have acceptable variations of non-lung organ at risks doses between the two plans to reduce bias. One of these plans will be randomly assigned using a 1:1 allocation ratio for the patient's treatment.

**Fig. 1:**
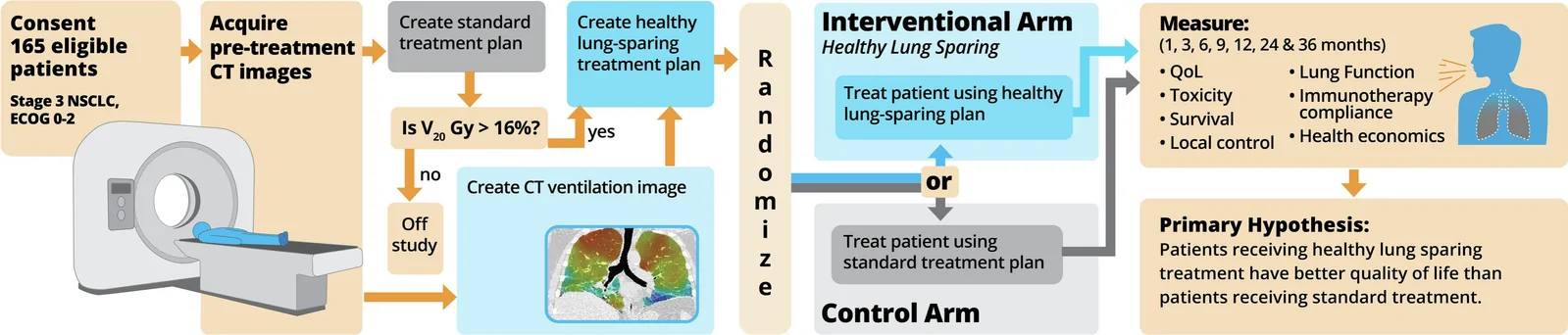
The double-masked randomized controlled VITaL phase 3 trial schema. VITaL will test the primary hypothesis of higher (better) QoL in the interventional healthy lung-sparing arm.

Treatment plans for both arms of the trial will follow the Cancer Institute New South Wales eviQ guidelines for respiratory NSCLC definitive external beam RT (https://www.eviq.org.au/radiation-oncology/respiratory/1871-respiratory-non-small-cell-lung-cancer-defini or https://www.eviq.org.au/radiation-oncology/respiratory/3697-respiratory-non-small-cell-lung-cancer-defini). The organs-at-risk will be contoured using the American Society for Radiation Oncology consensus guidelines [32]. The organs-at-risk dose constraints will use the same eviQ guidelines and follow the “as low as reasonably achievable principle”, minimizing dose to healthy tissue as reasonably achievable, whilst maintaining the treatment prescription. RT volumes and both plans will undergo standard departmental peer review. The randomly assigned plan (intervention or control) will be transferred to the treatment unit. There is no change in the treatment process between the two trial arms.

### 2.5 Randomization

Once the two plans are verbally approved by the treating oncologist, participants will be randomized. Those randomized to the interventional arm will be treated with the healthy lung-sparing plan. Those randomized to the control arm will be treated with the standard of care plan. The method of randomization will be minimization stratified by treating center, whether the patient will have concurrent chemotherapy, and whether the patient has planned adjuvant PD1/PD-L1 inhibitor treatment. To maintain double masking, both the healthy lung sparing plan and the standard of care plan will be approved by the treating physician prior to randomization. The treatment planner responsible for creating the plans is charged with plan upload, randomization, and sending the selected plan for treatment. The double masking for VITaL follows that used for the functional lung avoidance for individualized radiotherapy (FLAIR) trial [33]. All participants and investigators will be masked to the allocated treatment arm except the treatment planner responsible for exporting the selected plan, and any staff essential for the plan transfer, quality assurance processes.

### 2.6 Clinical and patient outcome measures

Patient assessments will occur at baseline, weekly during RT and 1, 3, 6, 9,12, 24 and 36 months after RT treatment. These assessments will include the physician-graded toxicity using the Common Terminology Criteria for Adverse Events (CTCAE v5.0), the FACT-L version 4 and local control using the Response Evaluation Criteria in Solid Tumors (RECIST v1.1) on follow up CT scans where available. FACT-L will be offered in multiple languages (Arabic, Chinese, English, Greek, Italian, Macedonian, Spanish, Turkish, and Vietnamese) to accommodate most primary spoken languages of patients at our recruiting sites.

### 2.7 Primary and secondary endpoints

The primary outcome, developed with consumer (people with lived cancer experience as a patient or carer trained to advance cancer research) input, was deemed relevant to consumers and aligned with Common Sense Radiation Oncology guidelines [34]. The primary hypothesis examines whether patients in the interventional healthy lung-sparing arm will experience a clinically meaningful better QoL over 3-months than patients receiving standard care. QoL will be measured using the FACT-L TOI at baseline and 3-months. FACT-L is a validated patient reported outcome (PRO) instrument available in several different languages measuring domains of physical, social/family, emotional and functional wellbeing and the TOI [30], [35], [36]. The TOI is a subscale combining Physical and Functional Well-being scales together with the lung cancer subscale. This provides relevant and patient-focused indicator of health-related QoL for the lung cancer patients participating in this clinical trial [35]. The threshold for a clinically meaningful difference in TOI decline is between 5 and 7 TOI units [37]. VITaL is powered to detect a decline of ≥5, the threshold used in the 424-patient Radiation Therapy Oncology Group (RTOG) 0617 trial [30].

The primary outcome measure will be evaluated at the 3-month timepoint to minimize the influence of immunotherapy toxicity. The average onset of pneumonitis for patients treated with post-RT immunotherapy is 2–3 months [7], [8], [9], [38].

Secondary outcome measures compared between the two arms include:

• Patient-reported outcomes at other timepoints using the physical, functional, social/family and emotional well-being FACT-L subscales.
• Clinical measures of respiratory, esophageal and cardiac toxicity at baseline, during treatment and at 1, 3, 6, 9, 12, 24 and 36-month follow up appointments using CTCAE v5.0 grading.
• Lung function changes using the pre- and post-treatment Forced Expiratory Volume in one second (FEV1) scores.
• The proportion of patients receiving and completing adjuvant immunotherapy.
• Overall survival, and time to disease progression at any site using RECIST, from randomization.
• A within-trial health economics analysis to compare the additional costs and health outcomes of healthy lung-sparing treatment compared with usual care from the perspective of the Australian healthcare system. We will conduct a cost-utility analysis to evaluate the additional cost per unit of health outcome gained. Utilities for quality adjusted life years (QALYs) will be scored from the self-reported Functional Assessment of Cancer Therapy – General (FACT-G) patient reported outcome instrument with an Australian value set, the FACT- eight dimension (FACT-8D) algorithm [39]. Costs will be measured in terms of direct costs to the health system over the 36-month study period. The economic outcome (or benefit) will be expressed as ‘per additional QALY gained’ after treatment. An incremental cost-effectiveness ratio (ICER) will be calculated for the additional cost per life years saved and cost per QALY gained [40].

### 2.8 Statistical considerations

The sample size estimates were derived from detecting a clinically meaningful difference of ≥5 points on the FACT-L TOI and using the measured QoL variance (10.5 units) [41]. Based on the *t-*test for comparing two means, a total sample size of 142 patients (71/group) will have 80% power with 95% two-sided confidence to detect a minimum 5-unit difference in mean TOI change score between the interventional healthy lung-sparing arm and the standard of care arm using 1:1 randomization. Assuming a 15% drop-out rate [42] prior to treatment due to upstaging or changes in the patient status, and loss of patients prior to the three-month assessment time point, 165 patients will be randomized.

## 3 Discussion

Sparing high-ventilated regions of the lung from higher radiation doses has shown reductions in treatment-related toxicity in non-randomized prospective and retrospective CTVI pilot trials [19], [20], [21], [22], [23], [24]. A 90-patient study concluded that dose to ventilated lung, identified using CTVI, is associated with pneumonitis, and RT should reduce dose to highly ventilated regions [29]. The 67-patient phase 2 trial NCT02528942 trial found that reducing dose to CTVI-identified high function regions reduced toxicity compared to historical rates and concluded that a phase 3 trial is warranted [25]. The 120-patient phase 2 NCT02843568 trial found that of the 60 patients treated with conventional fractionation, grade ≥ 2 pneumonitis was 8% with healthy lung-sparing vs. 32% without, suggesting a clinical benefit in patients with locally advanced NSCLC. Expanding the Faught et al. [19] US estimates globally, healthy lung-sparing RT will reduce the number of grade 2+ toxicities for 57,000+ global patients per year. These prior studies support the need for a phase 3 trial. The double-masked phase 3 randomized controlled Ventilation Imaging to reduce Toxicity for patients with Lung cancer (VITaL) trial will provide level 2 evidence for potential improvements in QoL, reductions in toxicity and increased eligibility to receive and complete a course of adjuvant immunotherapy. These findings may have broader applicability to other thoracic malignancies which require RT.

One distinguishing feature of the VITaL trial has been the active involvement of consumers since the conception of the trial. The consumers have consistently participated in and contributed to all Trial Management Committee meetings. Directly from consumer involvement the VITaL trial primary endpoint pivoted to patient reported QoL measures [43] from the initial clinically measured toxicity, which is now a secondary endpoint. QoL is associated with survival in lung cancer [44], [45], as QoL-limiting toxicities can lead to treatment discontinuation or indicate treatment ineffectiveness. Clinician reported toxicities can differ from patient reported outcomes, demonstrating the importance of directly asking the patient about their experience [46]. Feedback from our consumers in addition to the QoL focus has been positive regarding the support and engagement of the diverse national and international team; the involvement of early career researchers; the inclusion of regional cancer centers; and the inclusion of cancer centers with culturally and linguistically diverse patients.

## Abbreviations

**Table:** 

CTCAE	Common Terminology Criteria for Adverse Events
CT	Computed Tomography
CT:VQ	CT lung Ventilation and perfusion (Q) software
CTVI	Computed Tomography Ventilation Imaging
ECOG	Eastern Cooperative Oncology Group
FACT-8D	Functional Assessment of Cancer Therapy – Eight Dimension
FACT-G	Functional Assessment of Cancer Therapy – General
FACT-L	Functional Assessment of Cancer Therapy – Lung questionnaire
FEV1	Forced Expiratory Volume
FLAIR	Functional Lung avoidance for Individualized Radiotherapy
HREC	Human Research Ethics Committee
ICER	Incremental Cost-Effectiveness Ratio
NSCLC	Non-Small Cell Lung Cancer
PET	Positron Emission Tomography
PRO	Patient Reported Outcome
QALY	Quality Adjusted Life Years
QoL	Quality of Life
RECIST	Response Evaluation Criteria in Solid Tumors
RT	Radiation Therapy
TOI	Trial Outcome Index
TROG	Trans-Tasman Radiation Oncology Group
VITaL	Ventilation Imaging To improve the quality of Life for patients with lung cancer treated with RT

## Ethics approval and consent to participate

The Human Research Ethics Application 2023/ETH01802: *VITaL: A randomized controlled trial investigating Ventilation Imaging to reduce Toxicity for Lung cancer radiation therapy patients* was approved by the South Western Sydney Local Health District Human Research Ethics Committee. The full protocol (version 3.0) is provided in the Supplementary data. Written informed consent will be obtained from all participants by the participating site principal investigator or delegate as per ICH-GCP requirements, after being provided time to review the Participant Information Sheet and Consent Form.

## Funding

The VITaL trial is funded by the Australian Government National Health and Medical Research Council Clinical Trials and Cohort Studies grant APP2042777. Study development activities at the University of Sydney were supported by Cancer Institute NSW Translational Program Grant 2019/TPG2165. Paul Keall receives support from NHMRC APP 2041792. Patient capitation costs for New South Wales (NSW) patients are supported by Cancer Institute NSW.

## Planned Timeline

At time of submission the VITaL trial is open at seven Australian sites with international sites in development. Recruitment has commenced and it is estimated that full accrual will be reached by December 2029.

## Availability of data and materials

After study findings have been published, un-identifiable (non-coded, completely de-identified) data will be shared with researchers and/or at an external data repository for further scientific research. Information about data sharing will be provided to study participants in the Patient Information Sheet.

## Authors' contribution

The Trial Management Committee (TMC) designed the protocol. VG, HB, and PK, designed the statistical approach. KKS revised the health economics plan. RM-B designed the QoL study. HLB, NH, PK and the TMC designed the processes and quality assurance. HB and PK drafted this manuscript based on the TMC-developed protocol. All authors read and approved the manuscript.

## Declaration of competing interest

The authors declare the following financial interests/personal relationships which may be considered as potential competing interests: Paul Keall is an inventor on patent US7668357B2, and John Kipritidis and Paul Keall are inventors of CTVI software, that have been licensed to 4DMedical. Nina Eikelis is an employee of 4DMedical. This information is disclosed in the patient informed consent form and will be disclosed in publications and presentations arising from this trial. Nicholas Hardcastle receives research funding for unrelated work from Varian Medical Systems. Nicholas Hardcastle is a paid consultant of SeeTreat Medical.
