# Supplementary material for: Study protocol for the TROG 21.08 VITaL trial: a randomized controlled trial investigating ventilation imaging to improve the quality of life for patients with lung cancer treated with radiation therapy
Source: Clin Transl Radiat Oncol. 2026 Oct 8;62:101312. doi: 10.1016/j.ctro.2026.101312 (PMC13684938; doi:10.1016/j.ctro.2026.101312)
Supplement: Supplementary file 1 [file mmc1.pdf]

A randomised controlled trial investigating ventilation imaging to improve the quality of life for patients with lung cancer treated with radiation therapy

VITaL

Protocol Number: IX-2022-CT-VITAL

CONFIDENTIAL

This document is confidential and the property of The University of Sydney

No part of it may be transmitted, reproduced, published, or used without prior written authorisation from the institution.

STATEMENT OF COMPLIANCE

This document is a protocol for a clinical research study. The study will be conducted in compliance with all stipulations of this protocol, the conditions of ethics committee approval, the NHMRC National Statement on Ethical Conduct in Human Research (2007) and the Integrated Addendum to ICH E6(R1): Guideline for Good Clinical Practice ICH E6(R2) annotated with TGA comments, effective 09 Nov 2016.

THE UNIVERSITY OF SYDNEY

*A randomised controlled trial investigating **V**entilation **I**maging **T**o improve the quality of life for patients with lung cancer treated with radiation therapy (VITaL)*

Protocol Version 3.0 dated 8 May 2026

Contents

Administrative Information 4
2. Trial Registration 4
2.1 Registry 4
2.2 Trial Registration – Data Set 4
3. Protocol version 4
4. Funding 4
5. Roles and responsibilities 4
5.1: Contributorship 4
5.2: Sponsor contact information 8
5.3: Sponsor and funder 8
5.4: Committees 9
Introduction 10
6. Background and Rationale 10
6.1 Background 10
6.2 Choice of Comparators 13
7. Objectives 13
8. Trial Design 14
Methods: participants, interventions, outcomes 16
9. Study Setting 16
10. Eligibility Criteria 16
10.1 Inclusion criteria 16
10.1 Exclusion criteria 17
11. Interventions 17
11.1: Intervention and Control 17
11.2: Modifications 18
12. Outcomes 19

13. Participant Timeline 22
14. Sample Size 22
15. Recruitment 23
Methods: Assignment of interventions (for controlled trials) 24
16. Allocation 24
16.1: Sequence Generation and Concealment Mechanism 24
16.2 Implementation and blinding 24
Methods: Data collection, management, analysis 25
18. Data Collection Methods 25
18.1 25
19. Data Management 27
20. Statistical Methods 27
Methods: Monitoring 27
21. Data Monitoring 27
21.1 Formal Committee 27
21.2 Interim Analysis 27
22. Harms 27
22.1 Definitions 28
22.2 Assessment and Documentation of Adverse Events 28
22.3 Serious Adverse Event Reporting 29
USADEs 29
23. Auditing 29
Ethics and Dissemination 30
24. Research Ethics Approval 30
25. Protocol Amendments 30
26. Consent 30
26.1 Recruitment 30
26.2 Ancillary Studies 30
27. Confidentiality 31
28. Declaration of Interests 31
29. Access to Data 31
30. Ancillary and Post-Trial Care 31
31. Dissemination Policy 31

31.1 Trial Results ... 31
31.2 Authorship ... 32
31.3 Reproducible Research ... 32
32. References ... 32
Appendices ... 36

This template is based on the SPIRIT 2013 Statement. See doi: https://doi.org/10.1136/bmj.e7586 for a detailed explanation on each item.

Administrative Information

2. Trial Registration

2.1 Registry

This trial has been registered on ClinicalTrials.gov: NCT06127654 and ANZCTR.org.au

2.2 Trial Registration – Data Set

All items from the World Health Organization Trial Registration Data Set have been included in the above registrations.

3. Protocol version

2.0 22 July 2025

4. Funding

Seed funding from an NHMRC Investigator Grant and a CINSW Program Grant.
Trial funding from the NHMRC Clinical Trial and Cohort Studies grant scheme.
Additional site funding for NSW sites from the Cancer Institute NSW.

5. Roles and responsibilities

5.1: Contributorship

Principal Investigator

Name and Title: Professor Paul Keall
Position: Director, Image X Institute – University of Sydney
Address: Suite 201, Biomedical Building – C81, 1 Central Avenue, Australian Technology Park, Eveleigh, NSW, 2015
Telephone: (02) 8627 1159
Email: Paul.Keall@sydney.edu.au

Associate Investigators

Name and Title: Professor Shalini Vinod
Position: Senior Staff Specialist Radiation Oncologist, Liverpool and Macarthur Cancer Therapy Centres
Address: Liverpool Hospital NSW, 2170
Telephone: (02) 8738 9805
Email: Shalini.vinod@health.nsw.gov.au

Name and Title: Associate Professor Sashendra Senthi
Position: Radiation Oncologist and Victorian Cancer Agency Clinical Research Fellow, The Alfred Hospital
Address: 55 Commercial Rd, Melbourne VIC 3004
Telephone: (03) 9076 2000
Email: S.Senthi@alfred.org.au

Name and Title: Professor Ricky O'Brien
Position: Director Medical Imaging Facilities, School of Health and Biomedical Sciences
Address: STEM College, RMIT University, Bundoora VIC 3083
Building 201, Level 8, Room 11
Telephone: 0417 556 985
Email: ricky.obrien@rmit.edu.au

Name and Title: Dr Nicholas Hardcastle
Position: Medical Physicist, Peter MacCallum Cancer Centre
Address: 305 Grattan St, Melbourne, VIC, 3000
Telephone: (03) 8559 5000
Email: nick.hardcastle@petermac.org

Name and Title: Dr Mbathio Dieng
Position: Research Fellow, Sydney School of Public Health- University of Sydney
Address: Levels 4,5,6 K25 Medical Foundation Building, University of Sydney, NSW, 2006
Telephone: (02) 9562 5044
Email: Mbathio.dieng@sydney.edu.au

Name and Title: Dr Rebecca Mercieca-Bebber
Position: Quality of Life Research Fellow, NHMRC CTC
Address: Levels 4,5,6 K25 Medical Foundation Building, University of Sydney, NSW, 2006
Telephone: (02) 9562 5000
Email: rebecca.mercieca@sydney.edu.au

Name and Title: Professor Amit Sawant
Position: Vice Chair of Medical Physics in Radiation Oncology, University of Maryland
Address: Department of Radiation Oncology, Division of Medical Physics, University of Maryland School of Medicine, 22 S. Greene St, GGJ-01, Baltimore, MD 21201
Telephone: 410 328 1186
Email: asawant@som.umaryland.edu

Name and Title: A/Prof Surein Arulananda
Position: Interim Director of Medical Oncology, Thoracic Oncology Lead and Director of Clinical Trials, Monash Health
Address: Level 7, MHTP Building, Monash Health, 246 Clayton Road, Clayton VIC 3168, Australia
Telephone: 0421450499
Email: surein.arulananda@monashhealth.org

Name and Title: Dr Dasantha Jayamanne
Position: Radiation Oncologist, Northern Sydney Cancer Centre
Address: Royal North Shore Hospital, St Leonards, NSW, 2065
Telephone: (02) 8425 3000
Email: dasantha.jayamanne@health.nsw.gov.au

Name and Title: Associate Professor Tokihiro Yamamoto
Position: Clinical Medical Physicist, UC Davis Health Comprehensive Center
Address: University of California, Davis, 4501 X St, CA, 95817, USA
Telephone: (+1) 916 734 0604
Email: toyamamoto@ucdavis.edu

Name and Title: Assistant Professor Bilal Tahir
Position: Yorkshire Cancer Research Senior Fellow, University of Sheffield
Address: The University of Sheffield Medical School, Beech Hill Rd, Sheffield, S10 2RX, UK
Telephone: (+44) 114 222 2000
Email: b.tahir@sheffield.ac.uk

Name and Title: Professor Rachael Morton
Position: Director of Health Economics and Health Technology Assessment, NHMRC Clinical Trials Centre, University of Sydney
Address: Levels 4,5,6 K25 Medical Foundation Building, University of Sydney, NSW, 2006
Telephone: (02) 9562 5013
Email: rachael.morton@sydney.edu.au

Name and Title: Dr John Kipritidis
Position: Medical Physicist, Northern Sydney Cancer Centre
Address: Royal North Shore Hospital, St Leonards, NSW, 2065
Telephone: (02) 8425 3000
Email: john.kipritidis@health.nsw.gov.au

Name and Title: Dr Hilary Byrne
Position: Program Manager, Image X Institute – University of Sydney
Address: Suite 201, Biomedical Building – C81, 1 Central Avenue, Australian Technology Park, Eveleigh, NSW, 2015
Telephone: (02) 8627 1106
Email: hilary.byrne@sydney.edu.au

Name and Title: Dr Cynleen Kai
Position: Radiation Oncologist, GenesisCare
Address: GenesisCare, Ground Floor, 230 Cooper Street, Epping, VIC 3076
Telephone: (03) 9409 8000
Email: Cynleen.Kai@genesiscare.com

Name and Title: Dr Monique Heinke
Position: Radiation Oncologist, GenesisCare
Address: GenesisCare St Vincent's Sydney – St Vincent's Hospital, 438 Victoria Street, Darlinghurst, NSW, 2010
Telephone: 0426 264 283
Email: Monique.Heinke@genesiscare.com

Name and Title: Dr Louise Nardone
Position: Radiation Oncologist, GenesisCare
Address: Gosford Hospital, Holden St, Gosford, NSW 2250
Email: Louise.Nardone@health.nsw.gov.au

Name and Title: Dr Jane Ludbrook
Position: Radiation Oncologist
Address: Calvary Mater Newcastle
Email: jane.ludbrook@calvarymater.org.au

Name and Title: Dr David Palma
Position: Radiation Oncologist [London Health Sciences Centre (LHSC)], Clinician-Scientist (Ontario Institute for Cancer Research), and Chair [Canadian Pulmonary Radiotherapy Investigators (CAPRI) Group].
Address: Verspeeten Family Cancer Centre, London, Ontario, Canada
Email: david.palma@lhsc.on.ca

Name and Title: Dr Houda Bahig
Position: Radiation Oncologist, University of Montreal Hospital Center (CHUM)
Address: CHUM, 1000 R. Saint-Denis, Montréal, QC H2X 0C1, Canada
Email: houda.bahig.med@ssss.gouv.qc.ca

Name and Title: Dr Benjamin Lok
Position: Clinician-Scientist (Princess Margaret Cancer Centre) and Associate Professor of Radiation Oncology (University of Toronto)
Address: Princess Margaret Cancer Centre, 200 Elizabeth Street, Toronto, Ontario M5G 2C4
Email: benjamin.lok@uhn.ca

Name and Title: Dr Yu Yang Soon
Position: Assistant Professor Medicine, National University Cancer Institute, Singapore
Address: National University of Singapore, 21 Lower Kent Ridge Road. Singapore 119077
Email: yuyang.soon@nus.edu.sg

Name and Title: Esther Sim
Position: Radiation Oncologist, GenesisCare
Address: St John Of God Berwick Specialist Centre, 55 Kangan Drive, Berwick, VIC, 3806
Email: Esther.Sim@genesiscare.com

Statistician
Name and Title: Professor Val Gebski
Position: Director, Biostatistics & Research Methodology – NHMRC Clinical Trial Centre, University of Sydney
Address: Levels 4,5,6 K25 Medical Foundation Building, University of Sydney, NSW, 2006
Telephone: (02) 9562 5000
Email: val.gebski@sydney.edu.au

Trial Coordination Centre
Image X Institute
University of Sydney, Suite 201, Biomedical Building – C81, 1 Central Avenue, Eveleigh, NSW, 2015

This trial has received official endorsement from the Thoracic Oncology Group Australasia (TOGA).

5.2: Sponsor contact information
The University of Sydney
Address: University of Sydney
Camperdown NSW 2006
Australia

5.3: Sponsor and funder
The University of Sydney will act as the sponsor of the trial. The University of Sydney is responsible for the overall conduct of the study, insurance and indemnity arrangements, and owns the study protocol and study data.

The University of Sydney will be responsible for the interpretation and analysis of the data, the writing of the report(s) and the decision to submit the report for publication and has the ultimate authority over these activities.

5.4: Committees

The Trial Management Committee (TMC) will meet monthly to manage the conduct of the trial and to discuss progress. The TMC members are listed in Table 1.

Table 1. The VITaL Trial Management Committee and roles.

**Name**	**Role**
Paul Keall	Trial Chair, Image X Institute
Shalini Vinod	Radiation Oncologist, Site Principal Investigator, Liverpool Hospital
Sasha Senthi	Radiation Oncologist, Site Principal Investigator, The Alfred Hospital
Val Gebski	Trial Statistician, NHMRC CTC
Ricky O'Brien	Director of Medical Imaging, RMIT
Nicholas Hardcastle	Site Medical Physicist, Peter MacCallum Cancer Centre
Mbathio Dieng	Health Economist, NHMRC CTC
Rebecca Mercieca-Bebber	Quality of Life Research Fellow, NHMRC CTC
Amit Sawant	Vice Chair of Medical Physics, University of Maryland
Surein Arulananda	Director of Medical Oncology, Monash Health
Dasantha Jayamanne	Radiation Oncologist, Site Principal Investigator, Royal North Shore Hospital and GenesisCare
Cynleen Kai	Radiation Oncologist, Site Principal Investigator, GenesisCare Epping, Vic
Esther Sim	Radiation Oncologist, Site Principal Investigator, GenesisCare Frankston, Vic
Andrew Bowen	Consumer
Miriam Cavanagh	Consumer
Lee Hunt	Consumer
Hilary Byrne	Medical Physics Research Fellow, Image X Institute
Rachael Morton	Director of Health Economics and Health Technology Assessment, NHMRC CTC
Tokihiro Yamamoto	Clinical Medical Physicist, UC Davis Health Comprehensive Center
Bilal Tahir	Yorkshire Cancer Research Senior Fellow, University of Sheffield
Esther Vicente	Research Associate, University of Maryland
John Kipritidis	Medical Physicist, Royal North Shore Hospital
Andreas Fouras	CEO, 4DMedical
Nina Eikelis	Director, Medical and Clinical Affairs, 4DMedical
Shona Silvester	Trial Coordinator, Image X Institute
Monique Heinke	Radiation Oncologist, GenesisCare
Louise Nardone	Radiation Oncologist, Gosford Hospital
Jane Ludbrook	Radiation Oncologist, Calvary Mater, Newcastle
Marcel Schulz	Trial Coordinator, Image X Institute
David Palma	Radiation Oncologist, Site Principal Investigator, Canadian Pulmonary Radiotherapy Investigators (CAPRI) Group
Houda Bahig	Radiation Oncologist, Site Principal Investigator, University of Montreal Hospital Center (CHUM)

Name	Role
Benjamin Lok	Clinician-Scientist, Site Principal Investigator, Princess Margaret Cancer Centre
Yu Yang Soon	Assistant Professor Medicine, Site Principal Investigator, National University Cancer Institute
Hang Min	Postdoctoral Research Fellow, Image X Institute

A subset of the TMC will form a Data and Safety Monitoring Sub-Committee (DSMSC) established by the Sponsor to assess at intervals the progress of the clinical trial, the safety data, and the critical efficacy endpoints, and to recommend to the sponsor whether to continue, modify, or stop the trial and consists of the study Principal Investigator (PI), Trial Medical Officer, Trial Medical Physicist, and a Trial Research Fellow. Only the Trial Medical Officer or treating clinician may make clinical trial-related medical decisions. The DSMSC will meet quarterly following the TMC meeting or more often if requested by the PI.

Introduction

6. Background and Rationale

6.1 Background

Lung cancer radiation therapy and pneumonitis

Lung cancer, the leading cause of cancer mortality and disability adjusted life years in the world[1] has a 5-year survival rate of only 25%.[2] Radiotherapy will benefit 77% of patients with a major role as curative treatment in stage III Non-Small Cell Lung Cancer (NSCLC).[3] Radiotherapy combats the cancer but also causes radiation-induced lung injury, deteriorating quality of life[4] and survival.[5] Pneumonitis is the most common radiotherapy side effect: with the standard of care adjuvant immunotherapy in patients with stage III NSCLC, the Grade 2+ rate of pneumonitis is 23%-44%,[5-9] is greater in the Asian population, and in older patients aged 65+ years[10]. A multicentre study found 86% of pneumonitis was attributable to radiotherapy, with 11% related to both radiotherapy and immunotherapy.[7]

Pneumonitis impacts the ability to receive adjuvant immunotherapy in locally advanced NSCLC, a treatment with significant survival benefit.[11] Pneumonitis is the commonest reason for not commencing, interrupting or ceasing immunotherapy early. 10-15% patients have to discontinue adjuvant immunotherapy due to pneumonitis[7,8,12] and this results in poorer survival.[13,14] The 4y overall survival rate was reduced from 60.2% for patients completing immunotherapy to 45.4% if immunotherapy was ceased due to pulmonary toxicities, including radiation pneumonitis.[14] Pneumonitis increases hospitalisation rates by 80%, emergency visits by 40% and medical costs by $8,600 per patient.[15] This increased toxicity drives the need for healthy lung sparing radiation therapy, i.e., the VITaL trial.

Introduction to CT ventilation imaging

The planning and delivery of RT is a balance between delivering a curative dose to the tumour while sparing healthy organs, such as the lungs, from collateral damage such as pneumonitis. To minimise radiation-induced lung injury, our team has invented[16] and pioneered ventilation imaging based on computed tomography (CT). This Australian-invented medical device,[16] now an international field of research,[17] uses CT scans routinely acquired for planning RT to compute a CT ventilation map showing high functioning and low functioning lung regions (Figure 1). This image is used as the basis for directing

radiation away from the healthy, high functioning regions towards the low functioning regions, thereby reducing toxicity and improving the patient's quality of life.

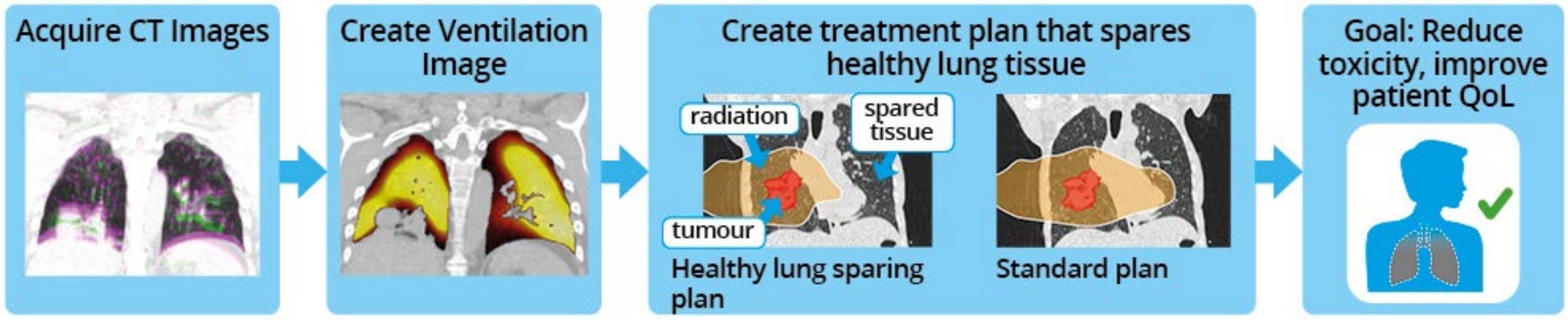

Figure 1. Ventilation imaging enables healthy lung to be identified and spared from high doses of radiation. The VITaL trial will test whether healthy lung sparing reduces toxicity.

Studies measuring the benefit of CT ventilation imaging

Evidence for the efficacy of the ventilation imaging method to reduce pneumonitis is detailed in references [17-20]. Non-randomised prospective and retrospective CT ventilation imaging pilot trials have shown that sparing high ventilated regions of the lung reduces treatment-related toxicity.[19,21-25]

Two systematic reviews have demonstrated the patient benefits of functional lung sparing.[17,20] The first systematic review[20] (114 included studies) proposed that future work was needed to detect differences between the anatomic and functional dose metrics. The second[17] (69 included studies) found moderate to strong evidence that CT ventilation imaging provides the same regional ventilation information as other modalities. The second review found 4 animal and 18 human studies comparing CT ventilation imaging to other modalities including xenon CT, SPECT (both DTPA and Technegas), PET and hyperpolarised MRI. The strength of correlation between CT ventilation imaging and the reference ventilation imaging modalities was moderate to strong when evaluated at the lobar or global level, with the average ± S.D. (number of studies) linear regression correlation coefficients were 0.73 ± 0.25 (n = 6) and 0.86 ± 0.11 (n = 12) for Jacobian-based and Hounsfield-unit-based CT ventilation imaging approaches respectively. These reviews logically lead to conducting this VITaL trial.

As CT scans are routinely acquired for lung cancer radiation therapy, CT ventilation imaging is low cost, easily implemented into the treatment workflow, and could therefore be made widely available. It is estimated that CT ventilation imaging will deliver major health benefits, reducing the risk of severe pneumonitis. Faught *et al*. [19] estimate that using CT ventilation imaging during radiation therapy will reduce severe pneumonitis in 5,200-8,300 patients per year in the US. Scaling globally, this estimate corresponds to reducing severe pneumonitis in >57,000 patients per year.

Prospective interventional CT ventilation imaging trials

Since CT ventilation imaging was invented in 2010,[16] several pilot trials have or are being undertaken, summarised in Table 2. These pilot trials have provided useful information to understand and clarify the role of CT ventilation imaging. The VITaL trial is a natural successor to the smaller pilot trials and will be the first to have reduced toxicity as the primary trial hypothesis.

Table 2. Summary of the pilot trials of CT ventilation imaging (CTVI) leading to the VITaL prospective randomised interventional trial. RT = radiation therapy.

Trial ID	Sponsor	Description/Primary endpoint	Key finding
NCT02528942	University of Colorado, NCI	Assessing safety and feasibility of CTVI RT using grade 2+ radiation pneumonitis	67-patient phase 2 trial concluding that a phase 3 trial is warranted.[26]
NCT02308709	UC Davis (AI Yamamoto)	Assessing safety and feasibility of CTVI RT using grade 3+ adverse events	CTVI functional avoidance RT with significantly reduced dose to ventilated lung regions could be safely administered.[27]
ACTRN 12612000775819	University of Sydney	Comparing CTVI to PET imaging	CTVI correlates with ground-truth PET ventilation.[28]
NCT02843568	University of Wisconsin	Assessing CTVI and lung tissue changes pre/post RT	Dose to healthy lung is associated with pneumonitis, radiation therapy should limit dose to functional regions.[29]

Together, the need for toxicity reduction, the early non-interventional analysis, the review papers, and the prospective pilot data all support the urgent need to conduct the VITaL trial and provide, for the first time, definitive evidence of the benefit – or lack thereof – of CT ventilation imaging for lung cancer patients.

6.2 Choice of Comparators

The control or comparator arm of the VITaL trial is the standard of care, where the ventilation of the lung is not known at the time of treatment planning, and therefore not used. The evidence-based, consensus driven cancer treatment protocols, the eviQ guidelines (https://www.eviq.org.au/), will be used volume delineation and radiotherapy dose prescription in both arms.

7. Objectives

Primary hypothesis: Patients receiving healthy lung sparing treatment (interventional arm) have better quality of life than patients receiving standard treatment (control arm).

Secondary hypotheses: In the interventional arm of healthy lung sparing treatment:

1. Patient will have improved quality of life at other timepoints and scales.
2. Patients will have reduced treatment lung side-effects.
3. Patients will have better lung function, as healthy lung is spared.
4. A higher proportion of patients will receive immunotherapy.
5. A higher proportion of patients will complete immunotherapy.
6. Cost effectiveness will be demonstrated as measured via a health economics assessment.
7. Patients will have improved survival.
8. Patients will have non-inferior local control.

Additional measures: Survival, progression-free survival, local control, patient reported functional, physical, social/family and emotional wellbeing scales, ventilation measures and treatment plan impacts on breath hold and 4DCT images.

8. Trial Design

Aim: The randomised controlled trial investigating Ventilation Imaging to reduce Toxicity for Lung cancer radiation therapy patients (VITaL) aims to improve quality of life and reduce treatment side-effects for patients with lung cancer (Figure 2). 165 patients will initially be recruited into the trial.

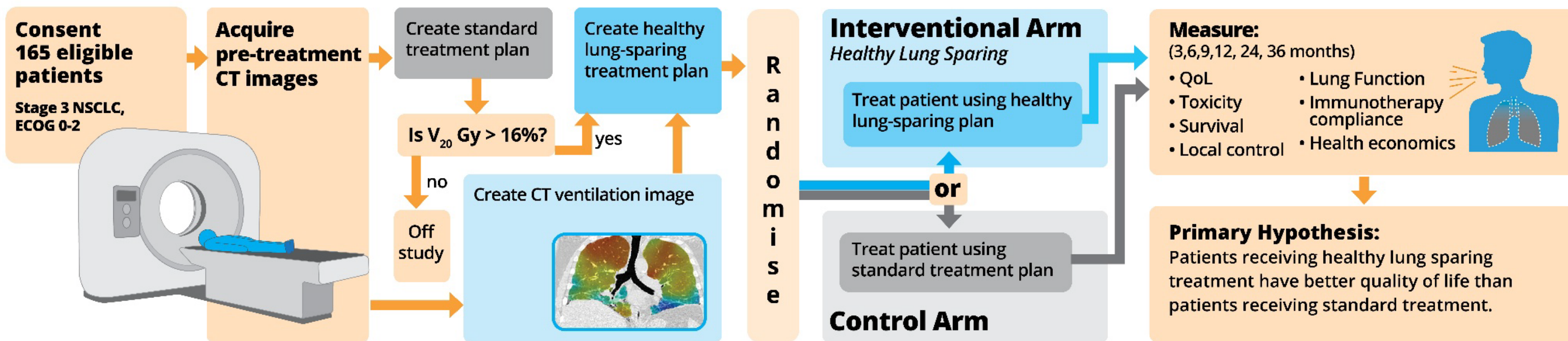

Figure 2. The double masked randomised controlled VITaL phase 3 trial schema. VITaL will test the patient-centred primary hypothesis, endorsed by our consumers, of higher quality of life in the interventional healthy lung-sparing arm.

How CT ventilation imaging can spare healthy lungs during radiation therapy is shown in Figure 3.

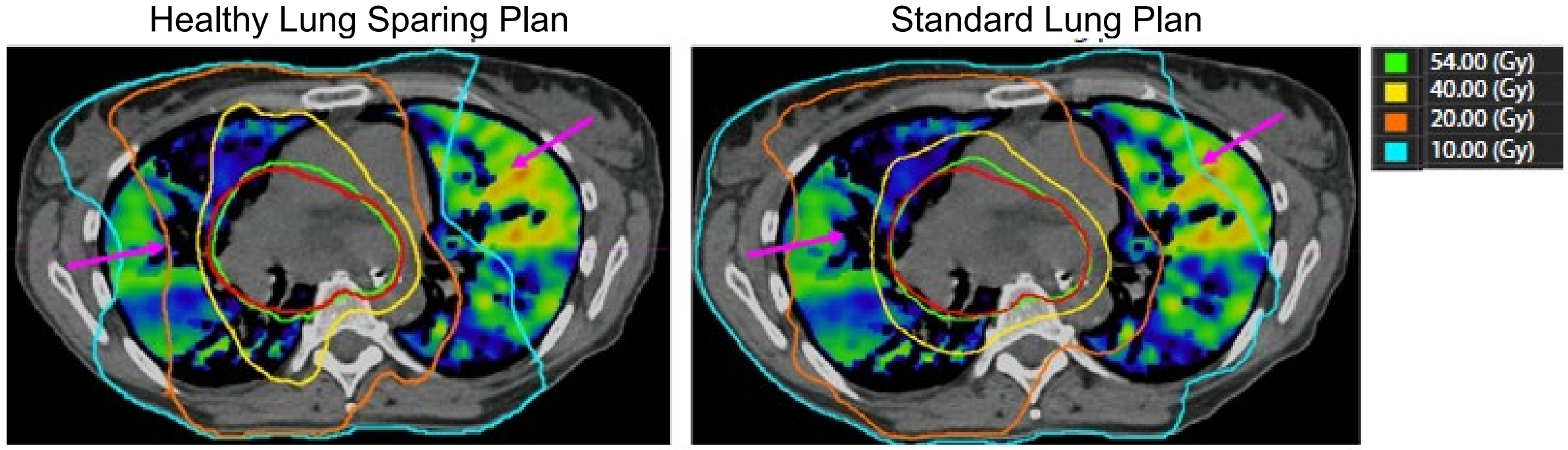

Figure 3. Ventilation imaging enables healthy lung (colourwash) to be identified and spared from high doses of radiation (blue, orange and yellow curves).[26] The heart, spinal cord and other critical organ doses are similar. The VITaL trial will test whether healthy lung sparing improves patient quality of life.

Methods: participants, interventions, outcomes

9. Study Setting

This trial will take place in cancer centres and hospitals in Australia. A full list of participating sites is in section 15.2

10. Eligibility Criteria

10.1 Inclusion criteria

The inclusion criteria for the VITaL trial are:

1. Aged 18 years or older.
2. ECOG performance status 0-2.
3. Histologically proven non-small cell lung cancer.
4. Stage 3 as determined using the IASLC (International Association for the Study of Lung Cancer) 8th edition lung cancer staging guidelines.
5. To be treated with curative intent external beam radiotherapy (60 Gy in 30 treatments or 55 Gy in 20 treatments) +/- concurrent chemotherapy and adjuvant PD1/PD-L1 inhibitors.
6. Whole body PET scan within 8 weeks of registration in study sites located in Australia.
   Whole body PET scan recommended within 8 weeks of registration for study sites located internationally (not in Australia), with an extended window of up to 12 weeks permitted.
7. Pulmonary function tests within 12 weeks of registration.
8. Willingness to give written informed consent.
9. Willingness to comply with the study procedures and visit requirements.
10. Available for follow up for a minimum of 12 months and up to 3 years.

Post-inclusion criteria includes the results of a QoL assessment and treatment plan assessment, neither of which are known prior to consent. The post-inclusion criteria for the VITaL trial are:

1. FACT-L TOI score of ≥5, the clinically meaningful difference[30]
2. The total lung volume difference between the inspiratory and expiratory CT images must be more than 500cc (or more than 10% of the expiration CT volume).

3. The standard treatment plan shows that at least 16% of volume of the lungs (minus the GTV) will receive more than 20 Gy. This criterion is based on the difference in pneumonitis risk between patients receiving below (~8%) and above (~34%) this threshold (Figure 1B, reference [31]). This criterion is estimated to exclude fewer than 25% of eligible patients.

The QoL assessment is unlikely to eliminate any patients but is included for patients where no clinically meaningful difference will be possible. The treatment plan assessment may exclude 20% of otherwise eligible patients.

10.1 Exclusion criteria

The exclusion criteria for the VITaL trial are:

1. Serious medical comorbidities that may contraindicate curative radiotherapy.
2. Inability to attend full course of radiotherapy or follow-up visits.
3. A current or former diagnosis of interstitial lung disease.
4. Prior history of lung cancer within 5 years.
5. Prior thoracic radiotherapy at any time.
6. Prior surgery for this cancer within a year.
7. Prior chemotherapy for this cancer.
8. Pregnant or lactating women.

11. Interventions

11.1: Intervention and Control

Intervention arm: As shown in Figure 2, in the intervention arm images an additional breath hold inhale and exhale CT scan pair acquired during the treatment planning session will be used to determine the high and low ventilating lung. From the breath hold CT images, a CT ventilation image is then created and imported into the treatment planning system. The addition of inhale/exhale CT scans to create the ventilation images is based on our prior findings.[28] The CT ventilation image highlights the healthy lung regions. The treatment planning system is directed to preferentially spare these healthy regions.

Control arm: As shown in Figure 2, in the control arm, CT ventilation imaging will not be used for planning, and the treatment planner and planning systems will be masked to the underlying ventilation image. As standard of care, the lung will be treated as having uniform function in its entirety.

Common approaches: Both arms of the trial will follow the TROG Radiation Therapy Planning, Delivery and Quality Assurance (QA) Guidelines. These guidelines outline the requirements for simulation, target volume contouring (as per evidence-based eviQ guidelineshttps://www.eviq.org.au/[12,32]) , organ-at-risk contouring, planning, treatment delivery and QA.
Both arms will follow the ALARA principle of minimising as much dose to healthy tissue as reasonably achievable, whilst maintaining the treatment prescription.

The assigned plan (intervention or control) will be transferred to the treatment unit by the responsible planner. There is no change to the treatment process between the two trial arms.

Critical steps: In radiation therapy planning, it is possible to spare some organs from radiation dose at the cost of giving more dose to others. This variable will necessarily be tightly controlled in the VITaL

trial based on approaches previously taken to ameliorate this known issue.[34] Dr Hardcastle will lead the development of the guidelines and automated tools for ensuring balance in the mean lung dose and dose to the target and critical organs in the intervention and control arms. The guidelines and automated tools will be developed for each of the participating sites.

Randomisation: Once the two plans are approved, patients will be randomised. Those randomised to the interventional arm will be treated with the healthy lung sparing plan. Those randomised to the control arm will be treated with the standard of care plan. The method of randomisation will be minimisation stratified by treating centre, whether the patient will have concurrent chemotherapy, and whether the patient has planned adjuvant PD1/PD-L1 inhibitor treatment. To maintain double masking, both the healthy lung sparing plan and the standard of care plan will be approved by the treating physician prior to randomisation. The treatment planner responsible for creating the plans is charged with plan upload, randomisation, and sending the selected plan for treatment. The double masking for VITaL follows that used for the NCT02002052 Functional Lung Avoidance for Individualized Radiotherapy (FLAIR) trial,[35,36] which investigated using the much less accessible hyperpolarised gas for ventilation imaging. All participants and investigators will be masked to the allocated treatment arm except the treatment planner responsible for exporting the selected plan, and any staff essential for the plan transfer, quality assurance processes.

Clinical and patient outcome measurements: Patient assessments will occur at baseline, weekly during radiation therapy and 3, 6, 9,12, 24 and 36 months after treatment. These assessments will include the physician-graded toxicity (CTCAE v5.0), the Functional Assessment of Cancer Therapy – Lung questionnaire (FACT-L v4) and local control (RECIST v1.1 criteria) on standard-of-care post-treatment CT scans.

COVID-19: This study will incorporate steps to safely and effectively conduct study activities during the COVID-19 pandemic. COVID-19 measures will remain in place until public health restrictions have been lifted, at which point, the PI may decide whether to retain none, some or all of the measures. Measures will ensure the safety of participants, research personnel, other hospital staff and visitors, ensure the study data remains valid, and not cause undue burden to the health system.
Guidelines from local, state and federal governments will be adhered to, along with guidelines from Site Governance and the Sponsor. The Australian Government DoH and NHMRC's Guidance on clinical trials for institutions, HRECs, researchers and sponsors will also be followed.

11.2: Modifications

In the case of a plan adaptation being required, the process of simulation, CT ventilation imaging, and planning will be repeated. TROG will be notified when the decision to adapt has been made. The treatment planner, solely unmasked to the arm the patient is randomised to, will complete the treatment plan.

12. Outcomes

Primary hypothesis: Patients in the interventional healthy lung sparing arm will maintain their 3-month quality of life more than patients receiving standard care by a clinically meaningful difference.

Measurement and testing of the primary hypothesis:

The primary endpoint is the change in the Functional Assessment of Cancer Therapy-Lung (FACT-L v4, Appendix 1) Trial Outcome Index (TOI) from before to 3-months after treatment. FACT-L is a widely used and validated PRO instrument that measures physical, social/family, emotional and functional wellbeing and the TOI.[30,37,38] The TOI is a FACT-L subscale that combines the Physical and Functional Well Being scales together with the Lung Cancer subscales and provides a highly relevant and patient-focused indicators of health-related quality of life for lung cancer patients participating in an oncology clinical trial.[37] The threshold for clinically meaningful difference in TOI decline was determined to be between 5 and 7 TOI units.[39] VITaL is powered to detect a decline of ≤ 5, the threshold used in the 424-patient RTOG 0617 trial.[30]

The primary outcome measure will be evaluated at the 3-month timepoint to remove the confounding influence of immunotherapy toxicity. The average onset of pneumonitis for patients treated with post-RT immunotherapy is 2-3 months.[7-9,40]

Secondary outcome measures to assess the longer-term PROs will be measured up to 36 months after treatment. In addition to the FACT-L TOI, the FACT-L and FACT-lung cancer subscale (LCS) measures will be evaluated. The PRO assessment timepoints mirror the recording of toxicity, survival and local control to span the critical clinical outcome measures.

Measurement and testing of the secondary hypotheses:

The secondary hypotheses measurement and testing methods are in Table 3. The study is powered for the primary quality of life assessment, a quasi-continuous measure. Treatment side effects are an ordinal measure, and receiving and competing immunotherapy, are binary measures for which larger sample sizes are typically needed to detect differences. Therefore, the secondary outcome data will be used to assess the magnitude of differences and the clinical significance of the differences, ultimately to determine if further investigation is warranted.

Table 3. Measurement and testing of the secondary hypotheses and additional measures.

Hypothesis: Healthy lung-sparing radiation therapy:	Measurements and evaluation methods
Improves quality of life at other timepoints and scales	FACT-L TOI measures at time points other than the primary hypothesis 3-month time point comparing the average change from baseline in each of the two arms with a two-sided unpaired *t* test, corrected for multiple hypothesis testing.<br>FACT-L and FACT-LCS measures comparing the average change from baseline in each of the two arms with a two-sided unpaired *t* test, corrected for multiple hypothesis testing.<br>The FACT-L functional, physical, social/family and emotional wellbeing scales will also be evaluated as exploratory endpoints.
Reduces lung treatment side-effects	Clinician measurement of respiratory, oesophageal and cardiac toxicity at baseline and 3, 6, 9, 12, 24 and 36 month follow up appointments using CTCAE v5.0 grading. Analysed using ordinal scale statistics.
Improves lung function	The difference between pre and post treatment FEV1 scores. Analysed using a two-sided unpaired *t* test.
Enables a higher proportion of patients to receive immunotherapy	Analysed using a two-sided *z* test for comparing two independent proportions.
Enables a higher proportion of patients to complete immunotherapy	Analysed using a two-sided *z* test for comparing two independent proportions.
Is cost effective as determined from a health economics assessment	Detailed separately below
Improves survival	The time from randomisation to death from any cause. Analysed using the Kaplan-Meier method.
Has non-inferior local control	The time from randomisation to disease progression at any site using the Response Evaluation Criteria in Solid Tumours (RECIST) version 1.1 criteria. Analysed using the Kaplan-Meier method.

Health economics assessment of resource use, costs and health-related quality of life

The economic evaluation will consist of a within-trial analysis to evaluate the additional costs and health outcomes of healthy lung sparing treatment compared with usual care from the perspective of the Australian healthcare system. We will conduct a cost-utility analysis (CUA) to evaluate the additional cost per unit of health outcome gained. Utilities for quality-of-life adjustment will be scored from the self-reported Functional Assessment of Cancer Therapy (FACT-G) patient reported outcome instrument with an Australian value set, the FACT-8D algorithm,[41] used to generate health utilities. Costs will be measured in terms of direct costs to the health system over the 36- month study period. Healthcare use will be identified and measured from study records and trial case report forms (for hospitalisations), and through linkage to Medicare claims data for outpatient visits (MBS) and prescribed medications (PBS). Australian unit costs will be applied to the healthcare use data (e.g., Australian Refined Diagnostic Related Groups (AR-DRG)), and Medicare scheduled fees. Other healthcare use that is not recorded in

MBS or PBS (e.g., hospital admissions, private health services, use of allied health therapies) will be identified and measured via trial records. Private non-medical health services (e.g., physiotherapy) will be valued at published standard rates, if available, or as reported by participants. The economic outcome (or benefit) is expressed as 'per additional quality adjusted life years (QALY) gained' at 3-months after treatment. Both costs and outcomes will be reported separately for each group following best practice methods[42] and an incremental cost-effectiveness ratio (ICER) will be calculated for the additional cost per life years saved and cost per QALY gained at 3-months after treatment. A cost-effectiveness acceptability curve will be presented showing the probability of the multimodal hub being cost-effective at different willingness to pay levels. Several sensitivity analyses will be undertaken to explore uncertainties surrounding key parameters in the economic evaluation such as age. The results for complete cost and health outcome data (i.e., those with no missing data) as well as a strict per-protocol analysis of the data will be provided to identify the impact of missing data on the analysis and any sensitivity to protocol violations.

Additional scientific investigations

VITaL will provide the substantial new scientific data and terabytes of patient images with a large, linked database of patient demographic and clinical data, anatomic and ventilation images, and treatment outcomes. Therefore, in addition to the primary and secondary outcomes of the VITaL trial, there will be a wealth of additional scientific investigations that can be performed on the data acquired from this study. Examples include:

- Put the data on The Cancer Imaging Archives (TCIA) for public use. PI has data from two trials on the TCIA.[28,43]
- Use the data to run an international imaging Grand Challenge (PI Keall, AI Kipritidis and AI Tahir have all led imaging challenges).[44-46]
- Combining ventilation with functional airway mapping provides a further projected benefit.[47,48]
- Comparing CT ventilation imaging from the inhale/exhale CT scans with ventilation images acquired during 4D CT scans.
- Combining the clinical outcomes and imaging data to refine the CT ventilation imaging method.
- Machine learning applications to create CT ventilation images.
- Radiomics and machine learning applications to analyse CT ventilation images.
- Knowledge-based planning development for healthy lung sparing radiation therapy.

Those studies deemed of highest scientific interest by the Trial Management Committee will be performed during the timeline of this grant. However, given our commitment to sharing the data to support additional scientific investigations, the examples described above, and many more not described, could be performed in future work by the VITaL team or independently by other research teams.

The statistical analysis is based on a modified intention to treat. As both trial arms are receiving radiation therapy, only patients who have received one or more of the planned radiation therapy sessions will be included in the analysis.

13. Participant Timeline

The participant timeline is shown in Table 4.

Table 4. The VITaL trial study visits and procedures schedule. ✓ = required. o = optional.

Visits / Process	Enrolment Visit	Pre treatment	Treatment (weekly)	Post treatment 1 month	3 mo	6 mo	9 mo	12 mo	24 mo	36 mo
Eligibility screening	✓									
Informed consent	✓									
CT scan*		✓		o	✓	o	o	o	o	o
Adverse Event & Serious Adverse Event Assessment		✓	✓	✓	✓	✓	✓	✓	✓	✓
FACT-L[37]		✓		✓	✓	✓	✓	✓	✓	✓
FEV1 scores DLCO scores		✓		o	✓	o	o	o	o	o
Cost Questionnaire						✓		✓		

* The pre-treatment CT scan will include additional inhale and exhale CT scans. The post-treatment CT scan will be that routinely acquired prior to immunotherapy (if acquired). All CT scan reports will be uploaded to the VITaL REDCap database. CT scan images are required to be uploaded to TROG at baseline and 3 months post-treatment.

14. Sample Size

Primary hypothesis: *Patients receiving healthy lung sparing treatment (interventional arm) have better quality of life than patients receiving standard treatment (control arm).*

The sample size estimates are based on detecting a clinically meaningful difference of ≥5 points on the FACT-L TOI and the variance measured quality of life results[36] reported in the FLAIR trial (10.5 units).[35] Using a two-sided *t* test for comparing two independent means with 1-1 randomisation, a total sample size of 142 patients (71/group) will have 80% power with 95% confidence to detect a minimum 5 unit difference in mean TOI change score between the interventional healthy lung sparing arm and the standard of care arm.

Assuming a 15% drop-out rate[49] prior to treatment due to upstaging or changes in the patient status, and loss of patients prior to the 3 month assessment time point, 165 patients will be randomised.

Given the VITaL trial sample size is based only on the 27 patients recruited into the FLAIR trial[36] that has a large amount of uncertainty, to maximise the investment in the VITaL trial, provide more confidence in the observed effects, whilst respecting that the results from the trial may be negative, the trial design enables a trial extension if the primary or secondary endpoints are near clinical significance.

The information contributing to the primary outcome and the corresponding sample size is based on the FLAIR[36] study comprising of a total of 27 patients. As this study is targeting a sample size > 6 times that in FLAIR, the uncertainty surrounding the estimates in this trial may be at variance to that observed in FLAIR. If the difference in the uncertainty is modest, the primary objective may still be achieved if a sample size is re-estimated close to completion of recruitment. This re-estimation would comprise an increase in sample size based on the pooled variance and not trigger a sample size reduction. After a minimum of 85% (145/165) of patients have been accrued into the trial and their 3-month TOI obtained, the variance of the TOI for all patients will be calculated, and, based on how consistent this variance is with the FLAIR variance, the sample size will be reassessed to determine whether if increased the study will yield a favourable result and/or an increase in the study power. This re-estimation will be conveyed to the IDSMC for consideration/comment as part of the trial conduct.

15. Recruitment

15.1 Number of Participants

165 participants will initially be randomised. As detailed in section 14. Sample Size; After a minimum of 85% (145/165) of patients have been accrued into the trial, the sample size will be reassessed to determine whether if increased, the study will yield a favourable result and/or an increase in the study power.

15.2 Study sites

The VITaL trial will engage cancer centres in Australia and overseas to recruit to the trial. The following sites are included:

Liverpool and Macarthur Cancer Therapy Centres, Liverpool Hospital, Liverpool

Bankstown-Lidcombe Hospital, Bankstown, NSW

Campbelltown Hospital, Campbelltown, NSW

Southwestern Sydney Local Health District (SWSLHD), NSW

Central Coast Local Health District (CCLHD), NSW

Northern Sydney Cancer Centre, Royal North Shore Hospital, St Leonards

The Alfred Hospital, 55 Commercial Rd, Melbourne

Peter MacCallum Cancer Centre, 305 Grattan St, Melbourne

GenesisCare Pty Ltd

Gosford Hospital, Holden St, Gosford

Calvary Mater Newcastle

Wyong Hospital, Wyong

Canberra Hospital, ACT

ICON Network

Sir Charles Gairdner Hospital, WA

CAPRI Network, Canada

Sheffield University, UK

University of Maryland, USA

Princess Margaret Cancer Centre, Canada

University of Montreal Hospital Center (CHUM), Montréal, QC H2X 0C1, Canada
The National University of Singapore, Singapore

ICON Cancer Centre Concord

15.3 Expected Duration of Study

Patient accrual is conservatively estimated to take three years for the 165 patients. The patients will be followed for a minimum of one year, and for up to three years. Analysis and manuscript preparation and publication will take one year. The trial is expected to take five years to complete.

Methods: Assignment of interventions (for controlled trials)

16. Allocation

16.1: Sequence Generation and Concealment Mechanism

Patients in the VITaL trial will be randomised and stratified using a secure web application hosted at the University of Sydney.

16.2 Implementation and masking

Participants will be enrolled by the clinical trials teams at the participating sites. To ensure double masking randomisation will be performed following that used in the FLAIR trial.[35,36]

Methods: Data collection, management, analysis

18. Data Collection Methods

18.1

An electronic list of identifying information (participant name and study ID) will be kept by the participating site PI, in a location separate to the study data, according to departmental policies and procedures. Following participant identification and consent, participating site study personnel will extract image files and apply de-identification procedures to ensure removal of any identifying information. Files will be coded with Study ID and will be re-identifiable by the participating site study team. Other (non-image file) data will be entered into a secure, password protected REDCap database maintained by the University of Sydney, coded with Study ID and year of birth.

18.2 Retention

Withdrawal from treatment or follow-up

A Trial Participant may discontinue trial treatment for any of the following reasons:

- Unacceptable toxicity
- Intercurrent illness which prevents further treatment
- Withdrawal of consent for treatment by participant
- Any alterations in the participant's condition which justifies the discontinuation of treatment in the investigator's opinion
- Unable to be treated in accordance with the protocol requirements, e.g. dose constraints for radiotherapy plan cannot be met

Discontinuation of treatment does not necessarily indicate withdrawal from the trial. Participants wishing to withdraw should speak to their treating physician to determine exactly what they wish to withdraw from. This request should be documented in the patient's medical record.

The distinction between discontinuation of treatment and withdrawal from the trial is shown by the following definitions:

Protocol Treatment Discontinuation

A participant would be considered to have discontinued treatment where trial related treatment is ceased according to the reason(s) outlined above. The participant may however still agree to further follow-up assessments as scheduled.

The participants' discontinuation of treatment must be documented in the medical records and transcribed onto the relevant eCRF.

Participants on the intervention arm who withdraw from trial treatment (but not the trial altogether) will not be unmasked, but will have their plan switched to the control plan by the treatment planner. All other staff involved in the trial will remain masked. These participants (as long as they agree) will continue to receive follow up visits as per the protocol.

Participants on the control arm who withdraw from trial treatment (but not the trial altogether) will not be unmasked, and will continue to receive standard treatment. These participants (as long as they agree) will continue to receive follow up visits as per the protocol.

Withdrawal from Trial

Trial Participants have the option to completely withdraw from the trial at any time without giving a reason. Total withdrawal would occur in the circumstance that the participant decides to completely withdraw from all treatment aspects of the trial, and does not agree to any further scheduled follow up assessments. The participants' total withdrawal must be documented in the medical records and transcribed onto the relevant eCRF. Patients who withdraw from the trial will receive the standard of care treatment with standard of care follow up. Per the consent form, the participant's data will be collected and analysed up until when they withdrew, however no further information will be collected from this participant for the purpose of this trial.

19. Data Management

Identifying participant list and coded study data will be stored at the participating site on password-protected drives provided by, and according to the policies and procedures of, the governing body of each participating site. Identifying data will not leave the participating site.

Coded (de-identified, re-identifiable) image files will be saved, securely transferred to the University of Sydney and stored on a departmental server with access limited to University of Sydney study personnel.

After study findings have been published, un-identifiable (non-coded, completely de-identified) data will be shared with researchers or at an external data repository for further scientific research.

Data confidentiality

Subject confidentiality is strictly held in trust by the participating investigators, research staff, and the sponsoring institution and their agents. The study protocol, documentation, data and all other information generated will be held in strict confidence. No information concerning the study or the data will be released to any unauthorised third party, without prior written approval of the chief and principal investigators or as required by law.

Data which identify any of the trial participants will not be revealed to anyone not directly involved in the trial.

Confidentiality will be adhered to at all times as per the Health Records and Information Privacy Act (NSW) 2002.

20. Statistical Methods

The statistical methods are detailed in the Outcomes section above.

Methods: Monitoring

21. Data Monitoring

21.1 Formal Committee

A Data and Safety Monitoring Sub-Committee will be used for the VITaL trial. The Sub-Committee will safeguard trial participants by assessing the safety and efficacy of the intervention being investigated; monitor the overall conduct of the trial to ensure high quality; and ensure that the trial meets its primary objectives.

21.2 Interim Analysis

No interim analysis is planned.

22. Harms

Adverse event reporting for the VITaL trial will follow the requirements of the National Health and Medical Research Council, Australian Health Ethics Committee (AHEC) Position Statement "Safety monitoring and reporting in clinical trials involving therapeutic goods" (Nov 2016).

It is important that Adverse Events (AEs) are monitored in the interest of participant safety. The investigator at each trial site is responsible for assessing and reporting AEs as part of routine clinical care and data collection. A subset of AEs will be classified as 'serious' and will require expedited reporting.

22.1 Definitions

Adverse Device Effect (ADE): Adverse event related to the use of an investigational medical device. Note: This definition includes adverse events resulting from insufficient or inadequate Instructions for Use, deployment, implantation, installation, or operation, or any malfunction of the investigational medical device. This definition includes any event resulting from use error or from intentional misuse of the investigational medical device.

Serious Adverse Event (SAE): SAEs in medical device trials being conducted under the CTN scheme are defined as any adverse event that:

a. led to death

b. led to serious deterioration in the health of the participant, that either resulted in:

- a life-threatening illness or injury, or
- a permanent impairment of a body structure or a body function, or
- in-patient or prolonged hospitalisation, or
- medical or surgical intervention to prevent life-threatening illness or injury or permanent impairment to a body structure of a body function

c. led to foetal distress, fetal death or a congenital abnormality or birth defect.

Note: Planned hospitalisation for a pre-existing condition, or a procedure required by the Clinical Investigation Plan, without serious deterioration in health, is not considered a serious adverse event.

Unanticipated Serious Adverse Device Effect (USADE): Serious adverse device effect which by its nature, incidence, severity or outcome has not been identified in the current version of the risk analysis report.

22.2 Assessment and Documentation of Adverse Events

All adverse events are to be reported between baseline and 3 years post treatment and must be graded according to the National Cancer Institute (NCI) Common Terminology Criteria for Adverse Events (CTCAE) version 5.0 by a study investigator. For this study, CTCAE will be used as well as clinical assessment to collect toxicity data.

All non-serious adverse events (including anticipated and unanticipated device related adverse events) shall be recorded in the trial database via adverse event eCRFs and made available to the TGA on request. Internal statistical analysis of these data shall be performed at the time specified in the protocol and the TGA, investigators and responsible HRECs will be advised of any safety issues which may emerge during this process.

22.3 Serious Adverse Event Reporting

SAEs are to be reported from the commencement of radiotherapy to 30 days after the last fraction of radiotherapy.

An event will not be considered an SAE if:

- Hospitalisation is due to administration of trial procedures
- Hospitalisation is due to placement of a permanent intravenous catheter
- Hospice placement for terminal care if required
- Hospitalisation is due to pre-trial scheduled elective surgery
- Convenience purposes (e.g. transportation difficulties)
- Out-patient hospitalisation for procedures such as: Convenience purposes (e.g. transportation difficulties)
- Death is due to cancer (this is considered 'expected' and is therefore not an SAE)
- Any new cancer is diagnosed (this is to be reported on relevant eCRF)

USADEs

If a USADE is identified, the investigator at the trial site must complete a Serious Adverse Event eCRF. The investigator is responsible for notifying responsible HRECs and Research Governance Officers of the SAE according to local ethical and regulatory guidelines.
All USADEs occurring in Australian participants must be reported to the Therapeutic Goods Administration:

- for fatal or life threatening Australian USADEs, no later than 7 calendar days after being made aware of the case, with any follow-up information within a further 8 calendar days
- for all other Australian USADEs, no later than 15 calendar days after being made aware of the case

Note: When determining whether a USADE has occurred, where the sponsor's causality assessment conflicts with the assessment made by the site investigator, the site investigator's assessment cannot be downgraded by the sponsor (i.e. altered from 'related' to 'not related'). In this case, if an investigator's judgment triggers the reporting of a USADE, the opinion of both the investigator and the sponsor should be provided with any report sent to the TGA.

23. Auditing

Data will be centrally monitored and cleaned by the trial coordinator. If an on-site monitoring visit is required, a representative from the Sponsor will contact the site to arrange the visit. Once a date has been confirmed, the site should ensure that full patient notes of participants selected for source data verification are available for monitoring. Sponsor staff conducting on-site monitoring will review essential documentation and carry out source data verification to confirm compliance with the clinical trial agreement and trial protocol. If any problems are detected during the course of the monitoring visit, the sponsor will work with the Principal Investigator or delegated individual to resolve issues and determine appropriate action.

Ethics and Dissemination

24. Research Ethics Approval

Ethics approval will be sought from the South Western Sydney Local Health District Human Research Ethics Committee.

25. Protocol Amendments

Any amendments to the protocol will first be approved by the Trial Management Committee, before being referred to the lead HREC for review and approval. Following HREC approval, local governance approval will be required at each site before the new protocol can be implemented. If the change directly impacts participants, an amendment to the Participant Information Sheet and Consent Form may be necessary, and re-consent of existing participants may be required.

26. Consent

26.1 Recruitment

Potential participants will be given a copy of both (1) the Participant Information Sheet and Consent Form (PICF) and (2) the Participant Consent Form for the release of Commonwealth health information provided by Services Australia to review with the Principal Investigator or their delegate. Written informed consent will be obtained from all participants by the participating site principal investigator or delegate as per ICH-GCP requirements, after being provided time to take the PICF home and consider the information and discuss it with their primary physician, friends or family. The Participant Consent Form for the release of Commonwealth health information provided by Services Australia will ask participants to provide consent for extraction of their Medicare Benefits Schedule (MBS) and Pharmaceutical Benefits Scheme (PBS) data from the date of consent until the 30/06/2030.

26.2 Ancillary Studies

After study completion, de-identified (non-coded, non-re-identifiable) data will be available to researchers for further scientific research. Information about data sharing will be provided to study participants in the Participant Information Sheet.

Aligned with the NHMRC's Open Access Policy and the Health Studies Australian National Data Asset (HeSANDA) program, the VITaL trial will make the de-identified trial data publicly available via The Cancer Imaging Archives (TCIA, https://www.cancerimagingarchive.net/). The TMC has clinical images from two trials on the TCIA.[28,43] The TMC also plan to use the images as a basis to run an international imaging grand challenge, similar to previously scientific grand challenges we have led such as the SPARE[44] and MATCH[46] challenges.

Data stored at the university: In order to download / decompress the stored, de-identified data, participating researchers will agree to the terms of use for the data, including that the data are not to be published or otherwise redistributed without the express consent of the original investigator(s).
Data stored at an external repository: de-identified study data may be provided to an external research data repository, archive or register so that it may be made publicly available for other scientific research. Study data that are provided to an external research data repository will be stored at and managed by the external repository. Data will only be shared with repositories whose function has been reviewed

and approved by an accredited Research Integrity/Ethics Committee/Board, under a Materials Transfer Agreement with the University.

27. Confidentiality

Subject confidentiality is strictly held in trust by the participating investigators, research staff, and the sponsoring institution and their agents. The study protocol, documentation, data and all other information generated will be held in strict confidence. No information concerning the study or the data will be released to any unauthorised third party, without prior written approval of the chief and principal investigators or as required by law. Data which identify any of the trial participants will not be revealed to anyone not directly involved in the trial. Confidentiality will be adhered to at all times as per the Health Records and Information Privacy Act (NSW) 2002.

28. Declaration of Interests

Paul Keall is an inventor on patent US7668357B2 related to CT ventilation imaging. John Kipritidis and Paul Keall are inventors on CT ventilation imaging software disclosed to the University of Sydney. This intellectual property has been licensed to 4D Medical. The University of Sydney and the inventors under the University of Sydney IP policy may benefit financially from this research project. This information will be disclosed in the patient informed consent form and in publication and presentations arising from this trial. This conflict is included as part of the approved conflict management plan that Paul Keall has with the University of Sydney. Andreas Fouras is the founder and a director of 4D Medical that may benefit financially from this research project.

29. Access to Data

The sponsor will retain the final trial dataset. In order to maximise the scientific and clinical use of data acquired in expensive and time-consuming clinical research, and in line with other archives, e.g., TCIA (https://www.cancerimagingarchive.net/), de-identified medical images and data from the VITaL trial are planned to be made publicly available.

30. Ancillary and Post-Trial Care

The University of Sydney endorses the principles of the Medicines Australia Guidelines for Compensation for Injury Resulting from Participation in a Clinical Trial. To provide protection for trial participants involved in University of Sydney Clinical Trials, University of Sydney maintains a clinical trials insurance policy.

31. Dissemination Policy

31.1 Trial Results

The VITaL trial will provide level 1 evidence of the benefit, or otherwise, of sparing healthy lungs during radiation therapy. As a double-masked randomised trial, the primary and select secondary outcomes are expected to be included in a high-impact publication. Other secondary outcomes, the health economics analysis, and additional scientific studies detailed in the project plan are planned for top discipline-specific journals.

31.2 Authorship
Authorship of publications will follow the International Committee of Medical Journal Editors guidelines (https://www.icmje.org/).

31.3 Reproducible Research
After study completion, de-identified (non-coded, non-re-identifiable) data will be available to researchers for further scientific research. Information about data sharing will be provided to study participants in the Patient Information Sheet. In order to maximise the scientific and clinical use of data acquired in expensive and time-consuming clinical research, and in line with other archives, e.g. TCIA (https://www.cancerimagingarchive.net/), de-identified medical images and data from the VITaL trial are planned to be made publicly available. They may also be used in imaging scientific challenges, similar to previously scientific grand challenges we have led such as the SPARE[44] and MATCH[46] challenges.

32. References

1. Kocarnik JM, Compton K, Dean FE, et al. Cancer incidence, mortality, years of life lost, years lived with disability, and disability-adjusted life years for 29 cancer groups from 2010 to 2019: a systematic analysis for the global burden of disease study 2019. *JAMA oncology.* 2022;8(3):420-444.
2. American Cancer Society. *Lung Cancer Survival Rates*. https://www.cancer.org/cancer/lung-cancer/detection-diagnosis-staging/survival-rates.html.
3. Barton MB, Jacob S, Shafiq J, et al. Estimating the demand for radiotherapy from the evidence: a review of changes from 2003 to 2012. *Radiother Oncol.* 2014;112(1):140-144.
4. Farr KP, Khalil AA, Grau C. Patient-reported lung symptoms and quality of life before and after radiation therapy for non-small cell lung cancer: correlation with radiation pneumonitis and functional imaging. *Acta Oncol.* 2019;58(10):1523-1527.
5. Altan M, Soto F, Xu T, et al. Pneumonitis After Concurrent Chemoradiation and Immune Checkpoint Inhibition in Patients with Locally Advanced Non-small Cell Lung Cancer. *Clin Oncol.* 2023.
6. Hassanzadeh C, Sita T, Savoor R, et al. Implications of pneumonitis after chemoradiation and durvalumab for locally advanced non-small cell lung cancer. *Journal of Thoracic Disease.* 2020;12(11):6690.
7. Saito G, Oya Y, Taniguchi Y, et al. Real-world survey of pneumonitis and its impact on durvalumab consolidation therapy in patients with non-small cell lung cancer who received chemoradiotherapy after durvalumab approval (HOPE-005/CRIMSON). *Lung Cancer.* 2021;161:86-93.
8. Shintani T, Kishi N, Matsuo Y, et al. Incidence and risk factors of symptomatic radiation pneumonitis in non–small-cell lung cancer patients treated with concurrent chemoradiotherapy and consolidation durvalumab. *Clinical Lung Cancer.* 2021;22(5):401-410.
9. Jang JY, Kim SS, Song SY, Kim YJ, Kim S-w, Choi EK. Radiation pneumonitis in patients with non-small-cell lung cancer receiving chemoradiotherapy and an immune checkpoint inhibitor: a retrospective study. *Radiat Oncol.* 2021;16(1):1-13.
10. Wang Y, Zhang T, Huang Y, et al. Real-world safety and efficacy of consolidation durvalumab after chemoradiation therapy for stage III non-small cell lung cancer: a systematic review and meta-analysis. *Int J Radiat Oncol Biol Phys.* 2022;112(5):1154-1164.

11. Spigel DR, Faivre-Finn C, Gray JE, et al. Five-year survival outcomes from the PACIFIC trial: durvalumab after chemoradiotherapy in stage III non–small-cell lung cancer. *J Clin Oncol.* 2022;40(12):1301.
12. Girard N, Bar J, Garrido P, et al. Treatment characteristics and real-world progression-free survival in patients with unresectable stage III NSCLC who received durvalumab after chemoradiotherapy: findings from the PACIFIC-R study. *Journal of Thoracic Oncology.* 2023;18(2):181-193.
13. Faehling M, Schumann C, Christopoulos P, et al. Durvalumab after definitive chemoradiotherapy in locally advanced unresectable non-small cell lung cancer (NSCLC): real-world data on survival and safety from the German expanded-access program (EAP). *Lung Cancer.* 2020;150:114-122.
14. Xu T, Wu L, Gandhi S, et al. Treatment-related pulmonary adverse events induced by chemoradiation and Durvalumab affect survival in locally advanced non-small cell lung cancer. *Radiother Oncol.* 2022;176:149-156.
15. Ryan KJ, Nero D, Feinberg BA, et al. Real-world incidence and cost of pneumonitis post-chemoradiotherapy for stage III non-small-cell lung cancer. *Future Oncology.* 2020;16(01):4303-4313.
16. Keall PJ, Joshi SC. Method and system for using computed tomography to test pulmonary function. *US Patent 7,668,357* 2010.
17. Hegi-Johnson F, de Ruysscher D, Keall P, et al. Imaging of regional ventilation: Is CT ventilation imaging the answer? A systematic review of the validation data. *Radiother Oncol.* 2019;137:175-185.
18. Vinogradskiy Y, Castillo R, Castillo E, et al. Results of a multi-institutional phase II clinical trial for 4DCT-ventilation functional avoidance thoracic radiotherapy [published online ahead of print 2021/11/13]. *Int J Radiat Oncol Biol Phys.* 2021. doi: 10.1016/j.ijrobp.2021.10.147.
19. Faught AM, Miyasaka Y, Kadoya N, et al. Evaluating the toxicity reduction with computed tomographic ventilation functional avoidance radiation therapy. *Int J Radiat Oncol Biol Phys.* 2017;99(2):325-333.
20. Bucknell NW, Hardcastle N, Bressel M, et al. Functional lung imaging in radiation therapy for lung cancer: a systematic review and meta-analysis. *Radiother Oncol.* 2018;129(2):196-208.
21. Faught AM, Yamamoto T, Castillo R, et al. Evaluating which dose-function metrics are most critical for functional-guided radiation therapy. *Int J Radiat Oncol Biol Phys.* 2017;99(1):202-209.
22. Seppenwoolde Y, De Jaeger K, Boersma LJ, Belderbos JS, Lebesque JV. Regional differences in lung radiosensitivity after radiotherapy for non–small-cell lung cancer. *Int J Radiat Oncol Biol Phys.* 2004;60(3):748-758.
23. Vinogradskiy Y, Castillo R, Castillo E, et al. Use of 4-dimensional computed tomography-based ventilation imaging to correlate lung dose and function with clinical outcomes. *Int J Radiat Oncol Biol Phys.* 2013;86(2):366-371.
24. Lan F, Jeudy J, Senan S, et al. Should regional ventilation function be considered during radiation treatment planning to prevent radiation-induced complications? *Med Phys.* 2016;43(9):5072-5079.
25. Munawar I, Yaremko BP, Craig J, et al. Intensity modulated radiotherapy of non-small-cell lung cancer incorporating SPECT ventilation imaging. *Med Phys.* 2010;37(4):1863-1872.
26. Vinogradskiy Y, Castillo R, Castillo E, et al. Results of a multi-institutional phase 2 clinical trial for 4DCT-ventilation functional avoidance thoracic radiation therapy. *Int J Radiat Oncol Biol Phys.* 2022;112(4):986-995.

27. Yamamoto T, Kabus S, Bal M, et al. Four-Dimensional Computed Tomography Ventilation Image Guided Lung Functional Avoidance Radiation Therapy: A Single-Arm Prospective Pilot Clinical Trial. *Int J Radiat Oncol Biol Phys.* 2022.
28. Eslick EM, Kipritidis J, Gradinscak D, et al. CT ventilation imaging derived from breath hold CT exhibits good regional accuracy with Galligas PET. *Radiother Oncol.* 2017.
29. Flakus MJ, Kent SP, Wallat EM, et al. Metrics of dose to highly ventilated lung are predictive of radiation-induced pneumonitis in lung cancer patients. *Radiother Oncol.* 2023.109553.
30. Movsas B, Hu C, Sloan J, et al. Quality of life analysis of a radiation dose–escalation study of patients with non–small-cell lung cancer: a secondary analysis of the radiation therapy oncology group 0617 randomized clinical trial. *JAMA oncology.* 2016;2(3):359-367.
31. Gao RW, Day CN, Nathan YY, et al. Dosimetric predictors of pneumonitis in locally advanced non-small cell lung cancer patients treated with chemoradiation followed by durvalumab. *Lung Cancer.* 2022;170:58-64.
32. Antonia SJ, Villegas A, Daniel D, et al. Overall survival with durvalumab after chemoradiotherapy in stage III NSCLC. *New England Journal of Medicine.* 2018;379(24):2342-2350.
33. Wright JL, Yom SS, Awan MJ, et al. Standardizing normal tissue contouring for radiation therapy treatment planning: an ASTRO consensus paper. *Pract Radiat Oncol.* 2019;9(2):65-72.
34. Yamamoto T, Kabus S, von Berg J, Lorenz C, Keall PJ. Impact of four-dimensional computed tomography pulmonary ventilation imaging-based functional avoidance for lung cancer radiotherapy [published online ahead of print 2010/07/22]. *Int J Radiat Oncol Biol Phys.* 2011;79(1):279-288.
35. Hoover DA, Capaldi DP, Sheikh K, et al. Functional lung avoidance for individualized radiotherapy (FLAIR): study protocol for a randomized, double-blind clinical trial. *BMC cancer.* 2014;14(1):1-10.
36. Yaremko B, Hoover D, Capaldi D, et al. Functional lung avoidance for individualized radiation therapy (FLAIR): results of a randomized, double-blind clinical trial. *International Journal of Radiation Oncology, Biology, Physics.* 2017;99(2):E507.
37. Cella DF, Bonomi AE, Lloyd SR, Tulsky DS, Kaplan E, Bonomi P. Reliability and validity of the Functional Assessment of Cancer Therapy—Lung (FACT-L) quality of life instrument. *Lung cancer.* 1995;12(3):199-220.
38. Devpura S, Feldman AM, Rusu SD, et al. An analysis of clinical toxic effects and quality of life as a function of radiation dose and volume after lung stereotactic body radiation therapy. *Adv Radiat Oncol.* 2021;6(6):100815.
39. Cella D, Eton DT, Fairclough DL, et al. What is a clinically meaningful change on the functional assessment of Cancer therapy–lung (FACT-L) questionnaire?: results from eastern cooperative oncology group (ECOG) study 5592. *Journal of clinical epidemiology.* 2002;55(3):285-295.
40. Mushonga M, Tsao M, Louie A, et al. Timing of Radiation Pneumonitis in Stage 3 Non-Small Cell Lung Cancer Patients by Receipt of Durvalumab. *International Journal of Radiation Oncology, Biology, Physics.* 2022;114(3):e383.
41. King MT, Norman R, Mercieca-Bebber R, et al. The functional assessment of cancer therapy eight dimension (FACT-8D), a multi-attribute utility instrument derived from the cancer-specific FACT-general (FACT-G) quality of life questionnaire: development and Australian value set. *Value in Health.* 2021;24(6):862-873.

42. Husereau D, Drummond M, Petrou S, et al. Consolidated health economic evaluation reporting standards (CHEERS) statement. *International journal of technology assessment in health care.* 2013;29(2):117-122.
43. Hugo GD, Weiss E, Sleeman WC, et al. A longitudinal four-dimensional computed tomography and cone beam computed tomography dataset for image-guided radiation therapy research in lung cancer. *Med Phys.* 2017;44(2):762-771.
44. Shieh CC, Gonzalez Y, Li B, et al. SPARE: Sparse-view reconstruction challenge for 4D cone-beam CT from a 1-min scan. *Med Phys.* 2019;46(9):3799-3811.
45. Kipritidis J, Tahir BA, Cazoulat G, et al. The VAMPIRE challenge: a multi-institutional validation study of CT ventilation imaging. *Med Phys.* 2019;46(3):1198-1217.
46. Mueller M, Poulsen P, Hansen R, et al. The markerless lung target tracking AAPM Grand Challenge (MATCH) results. *Med Phys.* 2022;49(2):1161-1180.
47. Vicente E, Modiri A, Kipritidis J, et al. Functionally weighted airway sparing (FWAS): a functional avoidance method for preserving post-treatment ventilation in lung radiotherapy. *Phys Med Biol.* 2020;65(16):165010.
48. Vicente EM, Modiri A, Kipritidis J, et al. Combining Serial and Parallel Functionality in Functional Lung Avoidance Radiation Therapy. *Int J Radiat Oncol Biol Phys.* 2022;113(2):456-468.
49. Gebert P, Schindel D, Frick J, Schenk L, Grittner U. Characteristics and patient-reported outcomes associated with dropout in severely affected oncological patients: an exploratory study. *BMC Medical Research Methodology.* 2021;21(1):1-11.

Appendices

Appendix I – FACT-L Questionnaire

FACT-L (Version 4)

Below is a list of statements that other people with your illness have said are important. **Please circle or mark one number per line to indicate your response as it applies to the past 7 days.**

	PHYSICAL WELL-BEING	Not at all	A little bit	Some-what	Quite a bit	Very much
GP1	I have a lack of energy	0	1	2	3	4
GP2	I have nausea	0	1	2	3	4
GP3	Because of my physical condition, I have trouble meeting the needs of my family	0	1	2	3	4
GP4	I have pain	0	1	2	3	4
GP5	I am bothered by side effects of treatment	0	1	2	3	4
GP6	I feel ill	0	1	2	3	4
GP7	I am forced to spend time in bed	0	1	2	3	4

	SOCIAL/FAMILY WELL-BEING	Not at all	A little bit	Some-what	Quite a bit	Very much
GS1	I feel close to my friends	0	1	2	3	4
GS2	I get emotional support from my family	0	1	2	3	4
GS3	I get support from my friends	0	1	2	3	4
GS4	My family has accepted my illness	0	1	2	3	4
GS5	I am satisfied with family communication about my illness	0	1	2	3	4
GS6	I feel close to my partner (or the person who is my main support)	0	1	2	3	4
Q1	*Regardless of your current level of sexual activity, please answer the following question. If you prefer not to answer it, please mark this box ☐ and go to the next section.*					
GS7	I am satisfied with my sex life	0	1	2	3	4

THE UNIVERSITY OF SYDNEY

FACT-L (Version 4)

Please circle or mark one number per line to indicate your response as it applies to the past 7 days.

	**EMOTIONAL WELL-BEING**	**Not at all**	**A little bit**	**Some-what**	**Quite a bit**	**Very much**
GE1	I feel sad	0	1	2	3	4
GE2	I am satisfied with how I am coping with my illness	0	1	2	3	4
GE3	I am losing hope in the fight against my illness	0	1	2	3	4
GE4	I feel nervous	0	1	2	3	4
GE5	I worry about dying	0	1	2	3	4
GE6	I worry that my condition will get worse	0	1	2	3	4

	**FUNCTIONAL WELL-BEING**	**Not at all**	**A little bit**	**Some-what**	**Quite a bit**	**Very much**
GF1	I am able to work (include work at home)	0	1	2	3	4
GF2	My work (include work at home) is fulfilling	0	1	2	3	4
GF3	I am able to enjoy life	0	1	2	3	4
GF4	I have accepted my illness	0	1	2	3	4
GF5	I am sleeping well	0	1	2	3	4
GF6	I am enjoying the things I usually do for fun	0	1	2	3	4
GF7	I am content with the quality of my life right now	0	1	2	3	4

THE UNIVERSITY OF SYDNEY

FACT-L (Version 4)

Please circle or mark one number per line to indicate your response as it applies to the <u>past 7 days</u>.

	**<u>ADDITIONAL CONCERNS</u>**	**Not at all**	**A little bit**	**Some-what**	**Quite a bit**	**Very much**
B1	I have been short of breath	0	1	2	3	4
C2	I am losing weight	0	1	2	3	4
L1	My thinking is clear	0	1	2	3	4
L2	I have been coughing	0	1	2	3	4
B5	I am bothered by hair loss	0	1	2	3	4
C6	I have a good appetite	0	1	2	3	4
L3	I feel tightness in my chest	0	1	2	3	4
L4	Breathing is easy for me	0	1	2	3	4
Q3	Have you ever smoked?<br>No ___ Yes ___ If yes:					
L5	I regret my smoking	0	1	2	3	4
